# Selbstkritische, multizentrische Analyse der Behandlungs- und Kodierqualität am Beispiel der Nephroureterektomie anhand von Abrechnungsdaten

**DOI:** 10.1007/s00120-025-02691-6

**Published:** 2025-10-09

**Authors:** Nici Markus Dreger, Christine Lenhart, Friedrich-Carl von Rundstedt, Thomas Steiner, Mark Schrader, Chris Protzel, Martin Friedrich, Wolfgang Jäger, Frank vom Dorp, Alexander Roosen, Olaf Reichelt, Sven Hohenstein, Stephan Degener

**Affiliations:** 1 Helios Universitätsklinikum Wuppertal, Heusnerstr. 40, 42283 Wuppertal, Deutschland; 2 Praxis Urologie am Malkasten, Düsseldorf, Deutschland; 3 Fakultät für Gesundheit, Private Universität Witten/Herdecke, Witten/Herdecke, Deutschland; 4 Private Universität Witten/Herdecke gGmbH, Witten, Deutschland; 5 Helios Klinikum Erfurt, Erfurt, Deutschland; 6 Helios Klinikum Berlin-Buch, Berlin, Deutschland; 7 Helios Klinikum Schwerin, Schwerin, Deutschland; 8 Helios Klinikum Krefeld, Krefeld, Deutschland; 9 Helios Klinikum Wiesbaden, Wiesbaden, Deutschland; 10 Helios Klinikum Duisburg, Duisburg, Deutschland; 11 Helios Klinikum Velbert, Velbert, Deutschland; 12 Helios Klinikum Aue, Aue, Deutschland; 13 Helios Health Institute GmbH, Berlin, Deutschland

**Keywords:** Urothelkarzinom des oberen Harntraktes, „Coronavirus disease 2019“, Evidenzlücke bei Real-World-Daten, COVID-19 Pandemie, Leitlinienadhärenz, Upper tract urothelial carcinoma, Coronavirus disease 2019, Real world evidence gap, COVID-19 pandemic, Guideline adherence

## Abstract

**Hintergrund:**

Urothelkarzinome des oberen Harntrakts (UTUC) sind selten und machen etwa 5–10 % aller urothelialen Karzinome aus. Im Gegensatz zu Urothelkarzinomen der Harnblase sind UTUC bei Diagnosestellung oft bereits invasiv. Die radikale Nephroureterektomie (RNU) ist der Standard für die Behandlung, wobei minimalinvasive Verfahren zunehmend an Bedeutung gewinnen. Ziel dieser Studie war es, die Entwicklung der RNU in Hinblick auf Fallzahl, Operationsmethode, Behandlungsqualität und Leitlinienadhärenz herauszuarbeiten.

**Material und Methoden:**

Die Studie basiert auf einer retrospektiven Analyse von G‑DRG-Abrechnungsdaten aus 87 Helios-Krankenhäusern in Deutschland für den Zeitraum 2016 bis 2022. Eingeschlossen wurden Patienten mit einer Hauptdiagnose UTUC, die sich einer RNU unterzogen hatten. Die Operationsverfahren wurden in offen-chirurgisch und minimalinvasiv (laparoskopisch oder roboterassistiert) unterteilt. Es wurden verschiedene Parameter wie Krankenhausverweildauer (KVD), Komplikationen und postoperative Maßnahmen analysiert. Eine Post-hoc-Umfrage der Kliniken diente der Validierung der Abrechnungsdaten.

**Ergebnisse:**

Insgesamt wurden 594 Patienten mit RNU behandelt. Der Anteil roboterassistierter RNU stieg kontinuierlich an, während die offen-chirurgischen Eingriffe abnahmen. Minimalinvasive Verfahren führten zu kürzeren Krankenhausaufenthalten (9,9 vs. 12,3 Tage; *p* < 0,001), weniger Komplikationen wie Blutungsanämien (12 % vs. 26 %; *p* < 0,001) und intensivmedizinischer Betreuung (57 % vs. 71 %; *p* < 0,001). Die Resektion der Blasenmanschette wurde seltener bei der minimalinvasiven RNU in der Kodierung erfasst (6,6 % vs. 46 %; *p* < 0,001). Die intravesikale Rezidivprophylaxe wurde unabhängig von der Operationsmethode nur bei einem geringen Anteil der Patienten kodiert (10 % vs. 6,9 %; *p* = 0,116), wobei die Post-hoc-Umfrage zeigte, dass die Blasenmanschettenresektion von 92 % und postoperative Instillation von 52 % der Helios-Kliniken durchgeführt werden.

**Schlussfolgerung:**

Roboterassistierte Operationen zeigten Vorteile hinsichtlich kürzerer Krankenhausaufenthalte und geringerer Komplikationsraten. Allerdings wurde eine unzureichende und verbesserungswürdige Leitlinienadhärenz bei der postoperativen Instillation festgestellt. Die Analyse zeigt eine deutliche Diskrepanz („real world evidence gap“) zwischen kodierten DRG-Daten und tatsächlicher klinischer Versorgung. Dieses Missverhältnis beeinflusst zentrale Struktur- und Leistungsentscheidungen im deutschen Gesundheitssystem und muss bei der Interpretation administrativer Datensätze dringend berücksichtigt werden.

**Graphic abstract:**

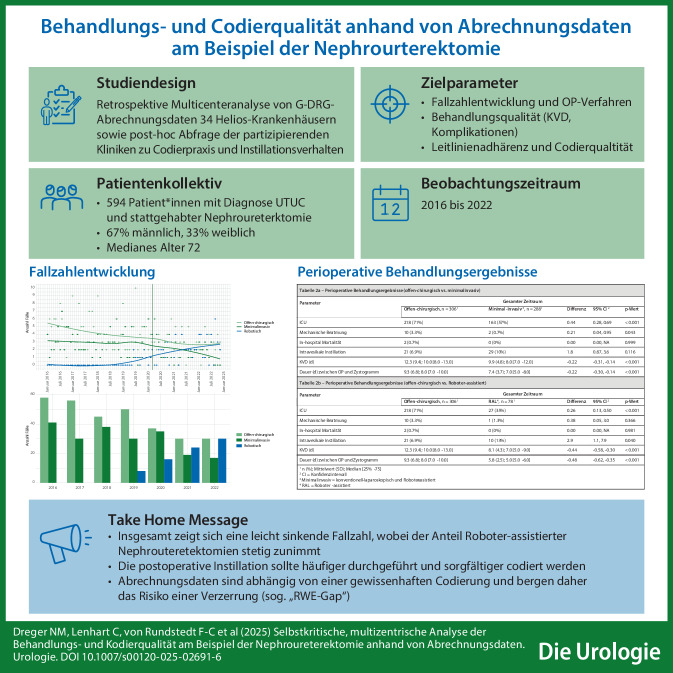

**Zusatzmaterial online:**

Die Online-Version dieses Beitrags (10.1007/s00120-025-02691-6) enthält weitere Tabellen.

## Abstract (en)

**Background:**

Urothelial carcinomas of the upper urinary tract (UTUC) are rare, accounting for approximately 5–10% of all urothelial carcinomas. In contrast to urothelial carcinomas of the bladder, UTUCs are often invasive at the time of diagnosis. Radical nephroureterectomy (RNU) is the gold standard for treatment, with minimally invasive procedures gaining increasing importance. Aim of this study was to examine the development of RNU in terms of case numbers, surgical methods, treatment quality, and guideline adherence.

**Materials and methods:**

This study is based on a retrospective analysis of German Diagnosis-Related Groups (G-DRG) billing data from 87 Helios hospitals in Germany for the period 2016–2022. Patients with a primary diagnosis of UTUC who had undergone RNU were included. Surgical procedures were classified as open surgery or minimally invasive (laparoscopic or robot-assisted). Various parameters such as length of hospital stay (LOS), complications and postoperative interventions were analyzed. A post hoc survey of the clinics served to validate billing data.

**Results:**

A total of 594 patients underwent RNU. The proportion of robot-assisted RNU steadily increased, while open procedures decreased. Minimally invasive procedures resulted in shorter hospital stays (9.9 days vs. 12.3 days; *p* < 0.001), fewer complications such as anemia due to bleeding (12% vs. 26%), and a decreasing need for intensive care (57% vs. 71%; *p* < 0.001). Bladder cuff resection was less frequently performed in minimally invasive RNU (6.6% vs. 46%; *p* < 0.001). However, intravesical instillation of chemotherapeutic agents was only performed in a small proportion of patients regardless of surgical method (10% vs. 6.9%; *p* = 0.116), although the post hoc survey revealed a coding bias and showed a cuff resection rate of 92% and an instillation rate of 52%.

**Conclusion:**

Robotic surgeries demonstrated significant advantages with regard to hospital stay and complications. However, insufficient guideline adherence was noted regarding postoperative instillation, highlighting points for improvement. The analysis shows a clear discrepancy (real-world evidence gap) between coded DRG data and actual clinical care. This disparity influences key structural and service decisions in the German healthcare system and must be urgently taken into account when interpreting administrative datasets.

## Einleitung

Mit einer Inzidenz von 1–2 Fällen pro 100.000 sind Urothelkarzinome des oberen Harntraktes (UTUC) selten, machen aber ca. 5–10 % aller urothelialen Karzinome aus [1]. Anders als beim Urothelkarzinom der Harnblase ist der Tumor des oberen Harntraktes zum Diagnosezeitpunkt bei etwa 60 % der Patienten invasiv wachsend, sodass es sich um ein Hochrisikokarzinom handelt [2]. In dieser Situation ist unabhängig von der Tumorlokalisation die radikale Nephroureterektomie (RNU) inklusive Blasenmanschette der Goldstandard. Ausnahmen können Karzinome des distalen Harnleiters sein. Die distale Ureterresektion in Kombination zu einer pelvinen Lymphknotendissektion kompromittiert das onkologische Ergebnis nicht. Ferner sind auch beim High-grade-UTUC organerhaltende Strategien bei Vorliegen imperativer Indikationen zu prüfen [3].

Die RNU kann offen oder minimalinvasiv durchgeführt werden. Der Trend geht zu minimalinvasiven Verfahren, auch wenn deren Überlegenheit nicht eindeutig nachgewiesen werden konnte [1, 4]. Die Leitlinie der European Association of Urology (EAU) empfiehlt, die RNU nicht länger als 12 Wochen nach Diagnose aufzuschieben, da sich dann das Progressionsrisiko erhöht [2]. Postoperativ sollte eine einmalige intravesikale Instillation mit einem Chemotherapeutikum erfolgen, da sich hierdurch die intravesikale Rezidivrate senken ließ [5].

Ziel dieser multizentrischen, deutschlandweiten Datenbankanalyse war, die Entwicklung der RNU in Hinblick auf Fallzahl, Operationsmethode, Behandlungsqualität und Leitlinienadhärenz herauszuarbeiten.

## Methode

### Datenerhebung

Als Datengrundlage dieser retrospektiven Studie dienten die in einer administrativen Datenbank (nach § 21 KHEntgG) vorliegenden G‑DRG-Abrechnungsdaten aller stationären Fälle der Krankenhäuser der Helios-Gruppe (*n* = 87). Betrachtet wurde der Zeitraum zwischen dem 1. Januar 2016 und dem 31. Dezember 2022 unter Berücksichtigung der COVID-19-Pandemie (mit 16.03.2020 als offiziellem Start des ersten Lockdowns).

Nur Patienten mit Hauptdiagnose *Urothelkarzinom des Nierenbeckens* (ICD-Code C65) und/oder *Urothelkarzinom des Harnleiters* (ICD-Code C66), die sich einer RNU (OPS-Code 5‑554.5) unterzogen haben, wurden eingeschlossen. Die Entfernung mit Blasenmanschette (OPS-Code 5‑575.0) wurde ebenfalls erfasst. Anhand der spezifischen OPS-Codes konnten die Patienten neben der oben genannten zeitlichen Einteilung auch anhand der verwendeten Operationstechnik eingeteilt werden in

- Offen-chirurgisch vs.
- minimalinvasiv (laparoskopisch und roboterassistiert), wobei durch den OPS-Zusatzcode 5‑978.0 diese Gruppe ebenfalls unterteilt werden konnte in laparoskopisch vs. roboterassistiert.

Für die Auswertung von Verlauf und Komplikationen wurden folgende Parameter herangezogen:

- die Intensivpflege (OPS 8‑980, 8‑98d, 8‑98f oder Dauer des intensivmedizinischen Aufenthalts > 0 Tage),
- mechanische Beatmung (OPS 8‑70x, 8‑71x oder Dauer der Beatmung > 0 Tage),
- Sterblichkeit im Krankenhaus (ausgenommen Patienten, die durch Verlegung ins Krankenhaus entlassen wurden) und
- Dauer des Aufenthalts (Nächte im Krankenhaus),
- zusätzliche Eingriffe am selben Tag oder im Verlauf,
- Diagnosen (Komplikationen),
- Instillation von und lokoregionale Therapie mit zytotoxischen Medikamenten und Immunmodulatoren in die Harnblase (OPS 8‑541.4; nach dem Haupteingriff),
- Tage zwischen Haupteingriff und Zystographie (OPS 3‑13f).

Die Analyse wurde von der Ethikkommission der Universität Leipzig bestätigt (eCaRe-COVID 19). Die Patientendaten wurden in anonymisierter Form gespeichert und die Datenverwendung wurde von der Datenschutzbehörde der Helios Kliniken GmbH genehmigt. Zudem wurde diese Studie durch die Helios Kliniken GmbH gefördert (Grant-ID: 2022-0315).

### Statistische Analyse

Die Verwaltungsdaten wurden aus QlikView (QlikTech, Radnor, PA, USA) extrahiert.

Inferenzstatistiken basierten auf verallgemeinerten linearen gemischten Modellen (GLMM) mit Krankenhäusern als Zufallsfaktor. Die Effekte wurden mit dem lme4-Paket (Version 1.1-26) in R‑Software für statistische Berechnungen (Version 4.0.2, 64-Bit-Build; R Core-Team 2020, https://www.r-project.org) geschätzt. In allen gemischten Modellen wurden variierende Achsenabschnitte für den Zufallsfaktor angegeben. Für alle Tests wurde ein Signifikanzniveau von 5 % festgesetzt.

Für die Beschreibung der Patientencharakteristika der Kohorten und der Komorbiditäten wurden χ^2^-Tests für kategoriale Variablen und t‑Tests mit zwei Stichproben für numerische Variablen verwendet.

Für den Vergleich der Anteile ausgewählter Behandlungen und Ergebnisse in den verschiedenen Kohorten haben wir logistische GLMM mit Logit-Link-Funktion verwendet.

Die Analyse der numerischen Variablen „Aufenthaltsdauer“ und „Tage zwischen dem Haupteingriff und der Zystographie“ wurde mit linearen gemischten Modellen durchgeführt. Da diese Variablen positiv verzerrt waren, transformierten wir sie mit Hilfe des inversen hyberbolischen Sinus, um eine annähernde Normalverteilung zu erhalten.

Für den gewichteten Elixhauser-Komorbiditätsindex wurde der AHRQ-Algorithmus angewendet.

Nachträglich führten wir anonymisiert eine Umfrage unter den partizipierenden Kliniken durch, um dem sog. „Real-World Evidence (RWE) Gap“ (auch „Discrepancy between Real-World Data [RWD] and Clinical Trial Data“ genannt) Rechnung zu tragen und keine falschen Schlussfolgerungen aus den Abrechnungsdaten zu ziehen. Diese Umfrage bestand aus 5 Fragen:

- Führen Sie in Ihrer Klinik bei der Nephroureterektomie standardmäßig eine Blasenmanschettenresektion durch?
- Falls Sie die Blasenmanschette standardmäßig resezieren: war Ihnen bewusst, dass es hierfür einen zusätzlichen OPS-Code (5–575.0) gibt?
- Führen Sie in Ihrer Klinik bei der Nephroureterektomie standardmäßig eine regionäre Lymphadenektomie (LND) durch?
- Führen Sie in Ihrer Klinik standardmäßig eine postoperative Instillation (bspw. mit Mitomycin C [MMC]) nach Nephroureterektomie durch?
- Falls Sie standardmäßig eine postoperative Instillation durchführen: wer ist für die Kodierung der Instillation verantwortlich?

## Ergebnisse

### Fallzahlentwicklung

Insgesamt wurden von 34 Helios-Zentren 594 Patienten mittels RNU behandelt. Abb. 2 zeigt die Fallzahlverteilung auf Monats- und Jahresebene, wobei mit Beginn der Pandemie nach Plateaubildung eine leicht rückläufige Anzahl zu beobachten war. Postpandemisch wurden präpandemische Fallzahlen nicht erreicht, wobei sich auch präpandemisch ein kontinuierlicher Rückgang (101 Fälle 2016 vs. 86 Fälle 2018) ergab. Der Tiefpunkt mit 74 RNU/Jahr wurde 2021 erreicht, was einer Reduktion von 26,7 % im Vergleich zu 2016 entspricht.

**Abb. 1:**
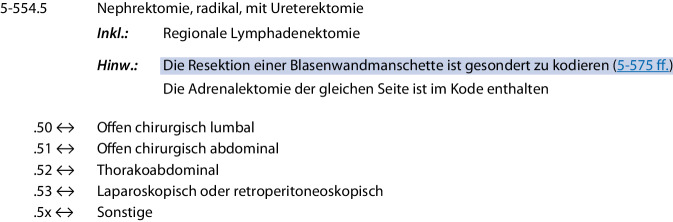
Operationen- und Prozedurenschlüssel (OPS) für die Nephroureterektomie gemäß Bundesministerium für Gesundheit (BfArM; [17])

**Abb. 2:**
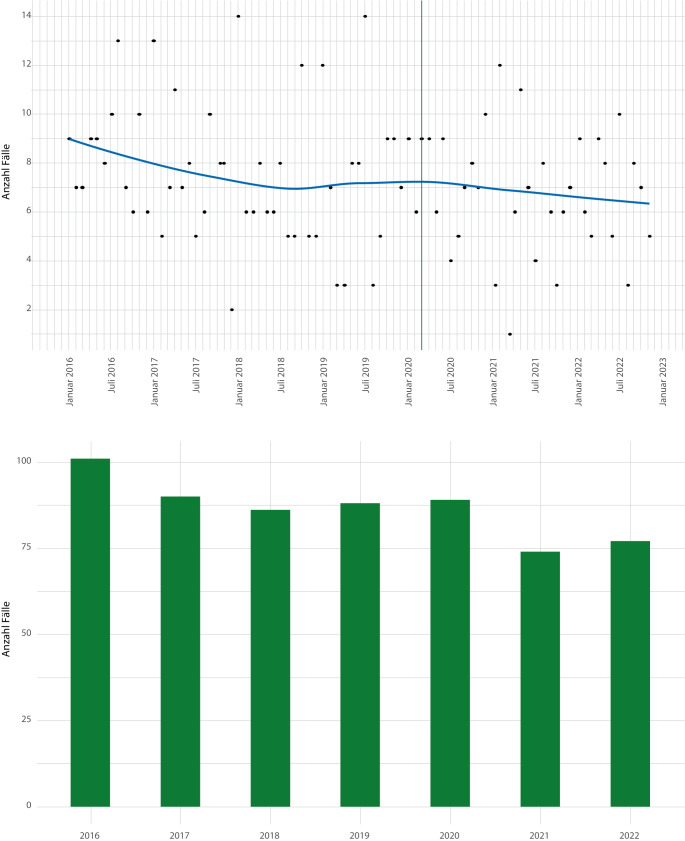
Fallzahlverteilung während des Beobachtungszeitraums auf Monatsebene (*obere Bildhälfte*: Die *senkrechte gestrichelte Linie *markiert den Zeitpunkt des ersten Lockdowns) sowie auf Jahresebene (*untere Bildhälfte*)

**Abb. 3:**
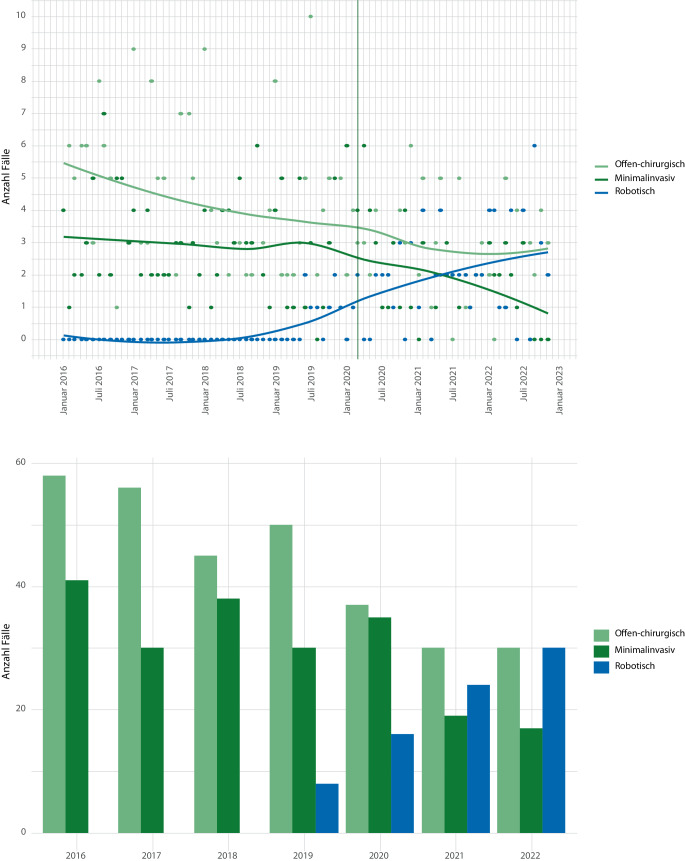
Verteilung der unterschiedlichen Operationsverfahren während des Beobachtungszeitraums auf Monats- (*obere Bildhälfte*) sowie auf Jahresebene (*untere Bildhälfte*)

Mit Blick auf das angewandte Operationsverfahren war nach Einführung roboterassistierter Operationssysteme Ende 2019 in einzelnen Helios-Kliniken ein kontinuierlich ansteigender Anteil der roboterassistierten laparoskopischen (RAL)-RNU zu erkennen, wohingegen die konventionell-laparoskopisch (LAP) durchgeführten RNU stetig abnahmen (Abb. 3). Über den gesamten Beobachtungszeitraum 2016–2022 wurde ebenfalls ein Abfall der offenen RNU beobachtet, wobei die Fallzahl 2021 und 2022 zumindest konstant blieb.

Von den insgesamt 594 ausgewerteten Patienten wurden 306 offen und 288 minimalinvasiv (210 LAP und 78 RAL) nephroureterektomiert (Tab. 1). Insgesamt erfolgte bei 140 (46 %) der Patienten aus der offen operierten und bei 19 (6,6 %) der Patienten aus der minimalinvasiven Gruppe die Entfernung einer Blasenmanschette, wobei der Anteil mit Blasenmanschette in der offen operierten Gruppe sukzessive abgenommen hat auf zuletzt 32 % (entsprechend 9 Patienten; Tab. 1).

**Tab. 1:** Patientencharakteristika

Parameter	Gesamter Zeitraum		
	Offen chirurgisch *n* = 306^a^	Minimalinvasiv^c^ *n* = 288^a^	*p*-Wert^b^
*Alter (Jahre)*	72,0 (10,2)	71,0 (10,1)	0,225
*Altersgruppe*			
< 18 Jahre	0 (0 %)	0 (0 %)	0,590
18–59 Jahre	38 (12 %)	36 (13 %)	
60–69 Jahre	77 (25 %)	84 (29 %)	
70–79 Jahre	115 (38 %)	108 (38 %)	
≥ 80 Jahre	76 (25 %)	60 (21 %)	
*Geschlecht*			
Männlich	208 (68 %)	189 (66 %)	0,603
Weiblich	98 (32 %)	99 (34 %)	
*Eingriff (OPS-Code)*			
5‑554,5 Nephrektomie, radikal, mit Ureterektomie	151 (49 %)	224 (78 %)	< 0,001
5‑554,6 Nephrektomie, radikal, mit endoskopischer Ureterexhairese	15 (4,9 %)	45 (16 %)	
5‑575,0 Partielle Harnblasenresektion: Teilresektion ohne Ureterneoimplantation (= Blasenmanschetten-resektion)	140 (46 %)	19 (6,6 %)	
*Offen chirurgisch*	306 (100 %)	0 (0 %)	< 0,001
*Konventionell laparoskopisch*	0 (0 %)	210 (73 %)	< 0,001
*Roboterassistiert*	0 (0 %)	78 (27 %)	< 0,001
*Elixhauser-Komorbiditätsscore*	16,2 (11,0)	11,9 (7,5)	< 0,001
*Elixhauser-Komorbiditätsindex*			
< 0	0 (0 %)	2 (0,7 %)	0,213
0	1 (0,3 %)	0 (0 %)	
1–4	17 (5,6 %)	23 (8,0 %)	
≥ 5	288 (94 %)	263 (91 %)	

^a^Mittelwert (SD); *n* (%)

^b^Welch Two Sample t‑Test; Pearson’s χ^2^-Test

^c^Minimalinvasiv = konventionell laparoskopisch und roboterassistiert

### Patientencharakteristika

Das durchschnittliche Alter zum Operationszeitpunkt lag zwischen 70,8 und 72,0 Jahren Die Gesamtkohorte verteilte sich zu knapp einem Drittel auf Frauen und zu zwei Dritteln auf Männer. Der zur Quantifizierung und besseren Vergleichbarkeit von Komorbidität bzw. Multimorbidität aus den Nebendiagnosecodes ermittelte Elixhauser-Komorbiditätsscore war signifikant höher in der offen operierten als in der minimalinvasiv operierten Kohorte. In absoluten Zahlen lag der Unterschied mit einem Delta von 4,3 Punkten (16,2 vs. 11,9 Punkte, *p* < 0,001; Tab. 1).

### Behandlungsparameter und Gruppenvergleiche

Mit Blick auf die Krankenhausverweildauer (KVD) ergab sich während des Beobachtungszeitraumes durchschnittlich ein Unterschied von 2,4 Tage (12,3 Tage offen chirurgisch vs. 9,9 Tage minimalinvasiv, *p* < 0,001; Tab. 2). In einer Subgruppenanalyse, bei der nur die RAL-RNU mit der offen-operierten Gruppe verglichen wurde, fiel dieser Unterschied noch größer aus mit einem durchschnittlich 4,2 Tage längeren Aufenthalt (12,3 vs. 8,1 Tage; *p* < 0,001) während des gesamten Beobachtungszeitraums (Tab. 3).

**Tab. 2:** Perioperative Behandlungsergebnisse (offen chirurgisch vs. minimalinvasiv)

Parameter	Gesamter Zeitraum				
	Offen chirurgisch *n* = 306^a^	Minimalinvasiv *n* = 288^a^	Differenz	95 %-KI	*p*-Wert
ICU	218 (71 %)	163 (57 %)	0,44	0,28, 0,69	< 0,001
Mechanische Beatmung	10 (3,3 %)	2 (0,7 %)	0,21	0,04, 0,95	0,043
In-hospital-Mortalität	2 (0,7 %)	0 (0 %)	0,00	0,00, NA	0,999
Intravesikale Instillation	21 (6,9 %)	29 (10 %)	1,8	0,87, 3,6	0,116
KVD (Tage)	12,3 (9,4); 10,0 [8,0–13,0]	9,9 (4,8); 8,0 [7,0–12,0]	−0,22	−0,31, −0,14	< 0,001
Dauer (Tage) zwischen Operation und Zystogramm	9,3 (6,8); 8,0 [7,0–10,0]	7,4 (3,7); 7,0 [5,0–8,0]	−0,22	−0,30, −0,14	< 0,001

*KI *Konfidenzintervall, *RAL *roboterassistiert, *minimalinvasiv *konventionell laparoskopisch und roboterassistiert, *KVD* Krankenhausverweildauer, *ICU* „intensive care unit“

^a^*n* (%); Mittelwert (SD); Median 25 %-75

**Tab. 3:** Perioperative Behandlungsergebnisse (offen chirurgisch vs. roboterassistiert)

Parameter	Gesamter Zeitraum				
	Offen-chirurgisch *n* = 306^a^	RAL *n* = 78^a^	Differenz	95 %-KI	*p*-Wert
ICU	218 (71 %)	27 (35 %)	0,26	0,13, 0,50	< 0,001
Mechanische Beatmung	10 (3,3 %)	1 (1,3 %)	0,38	0,05, 3,0	0,366
In-hospital Mortalität	2 (0,7 %)	0 (0 %)	0,00	0,00, NA	0,981
Intravesikale Instillation	21 (6,9 %)	10 (13 %)	2,9	1,1, 7,9	0,040
KVD (Tage)	12,3 (9,4); 10,0 [8,0–13,0]	8,1 (4,3); 7,0 [5,0–9,0]	−0,44	−0,58, −0,30	< 0,001
Dauer (Tage) zwischen Operation und Zystogramm	9,3 (6,8); 8,0 [7,0–10,0]	5,8 (2,5); 5,0 [5,0–6,0]	−0,48	−0,62, −0,35	< 0,001

*KI *Konfidenzintervall, *minimalinvasiv *konventionell laparoskopisch und roboterassistiert, *RAL *roboterassistiert, *KVD* Krankenhausverweildauer, *ICU* „intensive care unit“

^a^*n* (%); Mittelwert (SD); Median 25 %-75

Die Zeit zwischen Operation und Zystogramm unterschied sich signifikant: so betrug die Differenz im Mittel 1,9 Tage (9,3 Tage offen vs. 7,4 Tage minimalinvasiv; *p* < 0,001).

Die bereits in der Einleitung erwähnte intravesikale Instillationsprophylaxe wurde in der offen-operierten Kohorte nur bei 6,9 % (*n* = 21) und in der minimalinvasiven Kohorte bei 10 % (*n* = 29) angewandt (Tab. 2). In der Subgruppe der RAL-operierten Patienten erfolgte die Instillation signifikant häufiger (6,9 % vs. 13 %; *p* = 0,040).

Postoperativ mussten in der offen chirurgischen Gruppe 14 % mehr Patienten intensivmedizinisch überwacht werden als in der minimalinvasiven Gruppe (71 % vs. 57 % Patienten, *p* < 0,001; Tab. 2).

### Komplikationen

In *Ergänzungstabelle 1a* sind alle aufgetretenen peri- und postoperativen Komplikationen anhand der kodierten Diagnosen nach ICD-10 sowie notwendige Interventionen nach OPS-Code aufgelistet. Signifikante Unterschiede zwischen offen und minimalinvasiv operierten Patienten ergaben sich für die akute pulmonale Insuffizienz (4/306 [1,3 %] vs. 3/288 [1 %]; *p* = 0,031), die akute respiratorische Insuffizienz (27/306 [8,8 %] vs. 9/288 [3,1 %]; *p* = 0,005), die Zystitis (14/306 [4,6 %] vs. 5/288 [1,7 %]; *p* = 0,031), die Blutungsanämie (81/306 [26 %] vs. 34/288 [12 %]; *p* < 0,001) und das akute Nierenversagen Stadium III (18/306 [5,9 %] vs. 5/288 [1,7 %]; *p* = 0,016). Die Blutungsanämie war dabei signifikant häufiger transfusionspflichtig in der offen chirurgischen Kohorte (57/306 [19 %] vs. 11 [3,8 %]; *p* < 0,001), wobei in 2 Fällen der offenen RNU-Gruppe sogar 6–11 Erythroyztenkonzentrate transfundiert werden mussten (2/306 [0,7 %] vs. 0/288 [0 %]; *p* < 0,001). Das Signifikanzniveau knapp verfehlt haben „Hämatome als Komplikation nach OP“ (11/306 [3,6 %] vs. 3/288 [1 %]; *p* = 0,054) und „versehentliche Stich- oder Rissverletzungen während einer OP“ (26/306 [8,5 %] vs. 3/288 [1 %]; *p* = 0,064).

In der Subgruppenanalyse offen chirurgische vs. RAL-RNU wird für die genannten Komplikationen aufgrund der geringeren Gruppengröße zwar seltener das Signifikanzniveau erreicht, aber ein Blick auf die absoluten Zahlen zeigt deutliche Unterschiede in der Verteilung (Ergänzungstabelle 1b): akute pulmonale Insuffizienz 4/306 (1,3 %) vs. 0/78 (0; *p* = 1,000), akute respiratorisch Insuffizienz 27/306 (8,8 %) vs. 2/78 (2,6 %; *p* = 0,080), Zystitis 14/306 (4,6 %) vs. 2/78 (2,6 %; *p* = 0,214), Blutungsanämie 81/306 (26 %) vs. 8/78 (10 %; *p* = 0,002) und akutes Nierenversagen Stadium III (18/306 [5,9 %] vs. 1/78 [1,3 %]; *p* = 0,129). Analog zur minimalinvasiven Gruppe war auch in der RAL-RNU eine signifikant geringere Transfusionsrate nachweisbar mit 19 % vs. 6,4 % (57/306 vs. 5/78; *p* = 0,016).

Die SARS-CoV-2-Infektionen („severe acute respiratory syndrome coronavirus 2“) während des stationären Aufenthalts wurden nur bei insgesamt 3 Patienten beobachtet, ausnahmslos in der pandemischen Phase und in der Gruppe der offen chirurgischen RNU.

### Umfrage unter den partizipierenden Kliniken

Insgesamt 25/34 Kliniken nahmen an der Umfrage teil. Standardmäßig führen 23/25 Kliniken eine Blasenmanschettenresektion durch, wobei nur 9/25 Klinken angegeben haben, den zusätzlichen OPS-Code 5-575.0 hierfür gekannt zu haben. Eine LND führen 9/25 Kliniken „generell“ durch, 13/25 nur bei High-risk-UTUC gemäß EAU-Leitlinie und 3/25 verzichten auf eine LND. Die postoperative Instillation erfolgt standardmäßig in 13/25 Kliniken. Die Kodierung der Instillation erfolgt dabei in 7/25 Kliniken durch Assistenzärzt*innen, in 3/25 durch die Operateur*in und in 5/25 durch das Medizin-Controlling (11 Enthaltungen).

## Diskussion

Zwischen 2016 und 2022 hat sich die Anzahl der jährlich durchgeführten RNU in unserem Kollektiv verringert, wobei unmittelbar vor der Coronapandemie sowie danach Plateaubildungen zu verzeichnen waren. Auch wenn der Tiefpunkt 2021 mitten in die Coronapandemie fiel, zeigte sich 2019 und 2020 eine nahezu konstante Fallzahl. Eine Limitation unserer Arbeit ist, dass sie auf Abrechnungsdaten basiert, weshalb es uns nicht möglich war, etwaige Verzögerungen in der Diagnosestellung und Verschiebungen zu fortgeschritteneren onkologischen Stadien zu erfassen. Eine aktuelle Arbeit von Elleisy et al. stammt aus Deutschland und repräsentiert daher ähnliche gesundheitspolitische Rahmenbedingungen. Dabei wurden u. a. für die RNU keine Unterschiede in Bezug auf pathologische Stadien beobachtet [6].

Berücksichtigt man die Leitlinienempfehlung der EAU, ist die RNU standardmäßig mit Resektion der Blasenmanschette durchzuführen, um die Rate intravesikaler Rezidive zu reduzieren [2]. Große Kohortenstudien belegen eindeutig die onkologische Notwendigkeit der Blasenmanschettenresektion: Eine Mulitcenteranalyse von 820 Patienten der kanadischen RNU-Datenbank zeigte eine überlegene rezidivfreie 5‑Jahres-Überlebensrate (46,3 %) für die Manschettenresektion verglichen mit extravesikaler (35,6 %) und endoskopischer Technik (30,1 %; [7]). Die Samsung Medical Center-Studie mit 856 Patienten bestätigte, dass die Manschettenresektion der extravesikalen Ligatur deutlich überlegen ist mit einer signifikant besseren rezidivfreien 5‑Jahres-Überlebensrate (59,9 % vs. 49,3 %, *p* = 0,008) und einem besserem Gesamtüberleben (79,7 % vs. 68,0 %, *p* = 0,001; [8]). Eine aktuelle Metaanalyse von 2024 mit 1718 Patienten aus dem ROBUUST 2.0-Register untermauerte, dass die Blasenmanschettenresektion die intravesikale Rezidivfreiheitsrate verbessert, unabhängig von der verwendeten Technik [9]. Vor diesem Hintergrund überraschend war der in unserem Kollektiv geringe Anteil an Blasenmanschettenresektionen von 46 % in der offen chirurgischen Gruppe und v. a. der noch geringere Anteil von 6,6 % in der minimalinvasiven Gruppe. Im Vergleich zur offenen RNU ist bekannt, dass die Blasenmanschette bei LAP-RNU schwieriger sein kann. Mit Entwicklung der RAL-RNU wurde dieser Teilschritt deutlich erleichtert [9–11]. Hypothetisch wäre in der Subgruppe der RAL-RNU ein höherer Anteil mit Blasenmanschette resezierter Patienten zu erwarten gewesen – das Gegenteil war der Fall mit 3,8 %. Unser Verdacht, dass die wahrscheinlichste Erklärung hierfür eine unvollständige Operationskodierung ist, hat sich durch unsere Post-hoc-Umfrage unter den teilnehmenden Kliniken bestätigt: so haben 92 % angegeben, standardmäßige eine Blasenmanschettenresektion durchzuführen, aber 64 % davon hatten keine Kenntnis über den zu zusätzlich zu codierenden OPS-Code 5-575.0. Auch wenn dieser OPS-Code letztlich nicht erlösrelevant ist, wurde dies bereits selbstkritisch in der Helios-Fachgruppe diskutiert, da die Güte von Abrechnungsdaten und deren Analyse mit der Vollständigkeit der Kodierung korreliert. Unsere niedrige Kodierrate für die Manschettenresektion suggeriert daher fälschlich eine Leitlinien-Noncompliance und könnte Krankenversicherungen oder Aufsichtsbehörden gerade in Zeiten zunehmender Zentralisierung der Krankenhauslandschaft zu Fehlschlüssen verleiten.

Auch wenn die offene RNU insbesondere für fortgeschrittene Stadien als Standard in der EAU-Leitlinie gilt [2], findet sich in unserer Analyse eine stetige Zunahme der RAL-RNU zulasten der laparoskopischen wie auch der offenen RNU. Diese Entwicklung entspricht auch den aktuellen Studien, die eine Tendenz zu gleichwertigen onkologischen Ergebnissen nach RAL-RNU vs. offener RNU zeigen und dabei perioperative Vorteile bieten wie z. B. geringerer Blutverlust, und geringere Morbidität [4, 12–14]. In unserer Analyse zählten zu diesen perioperativen Benefits neben einer geringeren Anzahl transfusionspflichtiger Anämien (3,8 % vs. 19 %) auch eine seltenere Notwendigkeit intensivmedizinischer Betreuung (35 % vs. 71 %) oder mechanischer Beatmung (1,3 % vs. 3,3 %). Zudem verkürzte sich, analog zu den zitierten Studien, die KVD signifikant (8,1 vs. 12,3 Tage), was neben der angespannten Bettensituation während der Pandemie auch gesundheitsökonomisch als positive Entwicklung zu werten ist. Aus Sicht eines Operateurs ist das geringere Risiko für Nadelstichverletzungen hervorzuheben (0 % vs. 8,5 %). Der signifikant höhere Elixhauser-Komorbiditätsscore für die Gruppe der offenen RNU (16,2 vs. 11,9 Punkte) kann zum einen die klinische Praxis widerspiegeln, dass Patienten mit höherem Operationsrisiko häufiger dem „etablierten“ offenen Verfahren zugeführt werden. Zum anderen kann es ein Hinweis darauf sein, dass die Entscheidung des Operationsverfahrens in hohem Maße durch lokale Ressourcen (Robotikverfügbarkeit) und Lernkurveneffekte geprägt ist. Diese Erkenntnis relativiert pauschale Vergleiche zwischen „offen“ und „minimalinvasiv“ ohne Adjustierung für Tumorbiologie bzw. TNM-Stadium, was eine Limitation dieser rein auf Abrechnungsdaten basierenden Studie ist. Nichtsdestotrotz ist weder anhand der aktuellen Studien noch anhand dieser Daten die Frage eindeutig geklärt, welches Operationsverfahren überlegen ist.

Die in den Leitlinien empfohlene postoperative Instillation mit bspw. MMC kann unabhängig vom angewandten Operationsverfahren betrachtet werden und wurde in unserem Kollektiv bei 50/594 Patienten durchgeführt, entsprechend 8,4 %. Diese niedrige Rate wirft das Thema Leitlinienadhärenz auf. In der Literatur gibt es hierzu zwei Studien, die auf elektronischen Umfragen basieren: die Analyse von Dobe et al. ergab eine „regelmäßige“ Durchführung postoperativer Instillationen bei 47 % der Befragten, wobei die Antwortquote mit 11,6 % recht niedrig ausfiel. Bei den Befragten, die keine Instillation verabreicht haben, waren die häufigsten genannten Gründe das Fehlen unterstützender Daten (55 %), Angst vor möglichen Nebenwirkungen (18 %) und organisatorische Hürden (15 %; [15]). Die zweite Umfrage mit einer Antwortquote von 22 % ergab eine Instillationsrate von 51 %. Als Argumente gegen eine Instillation wurden ebenfalls das Fehlen unterstützender Daten (44 %) genannt sowie persönliche Präferenz (19 %) und infrastrukturelle Gründe (17 %; [16]). Betrachtet man nun die Ergebnisse unserer nachträglichen Umfrage, muss am ehesten eine insuffiziente Kodierung als Grund für die niedrige, aus Abrechnungsdaten erhobene Instillationsrate angenommen werden, da immerhin 52 % der Kliniken angegeben haben, diese standardmäßig durchzuführen und somit dem Niveau der beiden Studien entsprechen [15, 16]. Dass eine stattgehabte Instillation nicht kodiert wurde, kann begünstigt worden sein durch die heterogene Kodierverantwortlichkeit in den jeweiligen Kliniken (Operateur*in vs. Assistenzärzt*in vs. Medizin-Controlling). Unabhängig davon sollten diese Daten aber auch dazu dienen, im Sinne einer selbstkritischen Betrachtung die Leitlinienadhärenz zukünftig zu erhöhen.

Da der OPS-Code für die RNU per definitionem die regionale LND inkludiert (unabhängig davon, ob sie tatsächlich durchgeführt wurde oder nicht), wäre theoretisch eine 100 %-Rate an regionären LND in unserem Kollektiv anzunehmen. Dies entspricht aber nicht der Realität, wie unsere Umfrage zeigte, in der nur 36 % der Operateure eine regionale LND generell durchführen. Komplementär zur Blasenmanschettenresektion, bei der eine falsch-niedrige Rate durch Unkenntnis eines zusätzlichen OPS-Codes resultierte, suggerieren im Falle der LND reine Abrechnungsdaten eine falsch-hohe Rate und offenbaren somit eine weitere Schwäche OPS-Code basierter Analysen.

### Weitere Limitationen

In dieser Studie wurden Daten aus einer administrativen, multizentrischen Datenbank analysiert. Die Daten wurden nicht aus Forschungsinteressen, sondern aus Vergütungsgründen gespeichert, was die kodierten Informationen möglicherweise beeinflussen könnte. Zudem sind solche Daten abhängig von einer gewissenhaften Kodierung (s. Diskussion Blasenmanschette) – was nicht kodiert wurde, wird nicht erfasst und birgt das Risiko einer Verzerrung („RWE Gap“).

Ferner sei erwähnt, dass die Einführung neuer Operationsverfahren (roboterassistierte Operationssysteme) immer mit einer gewissen Lernkurve einhergehen und innerhalb der Helios-Gruppe auch nur schrittweise einzelne Kliniken mit diesen Systemen ausgestattet wurden. Die daraus resultierenden Lerneffekte gilt es bei der Interpretation solcher Daten zu berücksichtigen.

### Folgerungen zur Qualitätssicherung

- Einheitliche Kodierstandards müssen gruppenweit implementiert werden.
- Outcome-Indikatoren (Rezidiv- und Überlebensdaten) sollten – auch außerhalb von zertifizierten DKG-Zentren – prospektiv dokumentiert werden.
- Analysen administrativer Daten sind nur dann sinnvoll, wenn sie mit klinischen Real-world-Datensätzen rückgekoppelt werden.

## Schlussfolgerung

Die Kodierung operativer Eingriffe in Deutschland wird in hohem Maße durch die ökonomischen Anreize des DRG-Systems beeinflusst. Das Kodierergebnis spiegelt daher nicht zwingend das tatsächliche medizinische Vorgehen wider. Da in vielen Fällen keine alternativen Datensätze (z. B. Onkozert-Daten) vorliegen, besteht die Gefahr, dass Krankenkassen oder Aufsichtsbehörden falsche Rückschlüsse auf die tatsächliche Qualität der erbrachten medizinischen Versorgung ziehen.

Die von uns beobachtete, mangelnde Leitlinienadhärenz bzgl. der postoperativen Instillation sowie die nicht-standardisierte und heterogene Kodierung der Manschettenresektion als auch der Instillation müssen kritisch betrachtet werden und werden innerhalb der urologischen Fachgruppe bei Helios in Zukunft ein standardisierteres Vorgehen erforderlich machen.

## Supplementary Information

Peri- und postoperativen Komplikationen sowie notwendige Interventionen

## Data Availability

Die erhobenen Datensätze sind im Artikel oder im Zusatzmaterial online zu finden.
