# Supplementary material for: Self-critical, multicenter analysis of treatment and coding quality using the example of nephroureterectomy on the basis of billing data
Source: Urologie. 2025 Oct 9;65(3):288–97. [Article in German] doi: 10.1007/s00120-025-02691-6 (PMC12979303; doi:10.1007/s00120-025-02691-6)
Supplement: Supplementary file 1 — Peri- und postoperativen Komplikationen sowie notwendige Interventionen [file 120_2025_2691_MOESM1_ESM.pdf]

Zusatzmaterial
Selbstkritische, multizentrische Analyse der Behandlungs- und Kodierqualität am Beispiel der Nephroureterektomie anhand von Abrechnungsdaten.
Dreger et al. in „Die Urologie“

Ergänzungstabelle 1a – Postoperative Behandlungen und Komplikationen (offen-chirurgisch vs. minimalinvasiv)

Behandlung	Gesamter Zeitraum				
	Offen-chirurgisch, n = 306 [1]	Minimal-invasiv [3], n = 288 [1]	Differenz	95% CI [2]	p-Wert
5-408.2 Drainage einer Lymphozele	1 (0.3%)	0 (0%)	0.00	0.00, NA	0.999
5-462 Anlegen eines Enterostomas (protektiv)	1 (0.3%)	0 (0%)	0.00	0.00, NA	0.998
5-536 Verschluss Narbenhernie	2 (0.7%)	0 (0%)	0.00	0.00, NA	0.999
5-541 Laparotomie / Eröffnung des Retroperitoneums	5 (1.6%)	5 (1.7%)	1.1	0.30, 3.7	0.923
5-58 Operationen an der Urethra	0 (0%)	1 (0.3%)			
5-896 Chirurgische Wundtoilette [Wunddebridement] mit Entfernung von erkranktem Gewebe an Haut und Unterhaut	3 (1.0%)	0 (0%)	0.00	0.00, NA	0.924
5-896.0 Chirurgische Wundtoilette [Wunddebridement] mit Entfernung von erkranktem Gewebe an Haut und Unterhaut: Kleinflächig	1 (0.3%)	0 (0%)	0.00	0.00, NA	0.999
5-896.1 Chirurgische Wundtoilette [Wunddebridement] mit Entfernung von erkranktem Gewebe an Haut und Unterhaut: Großflächig	2 (0.7%)	0 (0%)	0.00	0.00, NA	0.954
5-896.1 Chirurgische Wundtoilette [Wunddebridement] mit Entfernung von erkranktem Gewebe an Haut und Unterhaut: Großflächig, mit Einlegen eines Medikamententrägers	2 (0.7%)	0 (0%)	0.00	0.00, NA	0.954
5-916.a Anlage oder Wechsel eines Systems zur Vakuumtherapie	4 (1.3%)	0 (0%)	0.00	0.00, NA	0.998
5-916.a5 Anlage oder Wechsel eines Systems zur Vakuumtherapie: Tiefreichend subfaszial an der Bauchwand oder im Bereich von Nähten der Faszien bzw. des Peritoneums	2 (0.7%)	0 (0%)	0.00	0.00, NA	0.989
8-148 Therapeutische Drainage von anderen Organen und Geweben	3 (1.0%)	3 (1.0%)	1.1	0.21, 5.3	0.941
8-148.0 Therapeutische Drainage von anderen Organen und Geweben: Peritonealraum	1 (0.3%)	2 (0.7%)	2.1	0.19, 24	0.537
8-148.1 Therapeutische Drainage von anderen Organen und Geweben: Retroperitonealraum inkl. Psoas	2 (0.7%)	1 (0.3%)	0.53	0.05, 5.9	0.605
8-800.c Erythrozytenkonzentrat	57 (19%)	11 (3.8%)	0.17	0.09, 0.34	< 0.001
8-800.c0 Erythrozytenkonzentrat: 1 TE bis unter 6 TE	53 (17%)	11 (3.8%)	0.19	0.10, 0.38	< 0.001
8-800.c1 Erythrozytenkonzentrat: 6 TE bis unter 11 TE	2 (0.7%)	0 (0%)	0.00	0.00, NA	0.964
8-713 Maschinelle Beatmung und Atemunterstützung bei Erwachsenen	1 (0.3%)	0 (0%)	0.00	0.00, NA	0.995
8-771 Kardiale oder kardiopulmonale Reanimation	2 (0.7%)	1 (0.3%)	0.53	0.05, 5.9	0.605
**Komplikationen**					
A40 Streptokokkensepsis	1 (0.3%)	0 (0%)	0.00	0.00, NA	1.000
A41 Sonstige Sepsis	10 (3.3%)	3 (1.0%)	0.35	0.09, 1.4	0.134
I21 Akuter Myokardinfarkt	4 (1.3%)	0 (0%)	0.00	0.00, NA	0.935
I26 Lungenembolie	2 (0.7%)	3 (1.0%)	1.6	0.27, 9.6	0.608
I63 Hirninfarkt	2 (0.7%)	0 (0%)	0.00	0.00, NA	0.999
I80 Thrombose, Phlebitis, Thrombophlebitis	5 (1.6%)	3 (1.0%)	0.24	0.03, 2.0	0.191
I82.2 Embolie und Thrombose der V. cava	1 (0.3%)	0 (0%)	0.00	0.00, NA	0.993
I82.8 Embolie und Thrombose sonstiger näher bezeichneter Venen	0 (0%)	1 (0.3%)	137,440	0.00, NA	0.961
I82.9 Embolie und Thrombose nicht näher bezeichneter Venen	1 (0.3%)	0 (0%)	0.00	0.00, NA	0.999
J95.2 Akute pulmonale Insuffizienz	4 (1.3%)	3 (1.0%)	0.07	0.01, 0.78	0.031
J96.0 Akute respiratorische Insuffizienz	27 (8.8%)	9 (3.1%)	0.33	0.15, 0.72	0.005
K25.0 Ulcus ventriculi: Akut, mit Blutung	2 (0.7%)	0 (0%)	0.00	0.00, NA	1.000
K26.0 Ulcus duodeni: Akut, mit Blutung	2 (0.7%)	1 (0.3%)	0.51	0.02, 15	0.695
K29.1 Sonstige akute Gastritis	0 (0%)	1 (0.3%)	157,145,914	0.00, NA	0.994
K65 Peritonitis	5 (1.6%)	0 (0%)	0.00	0.00, NA	0.920
K43.2 Narbenhernie ohne Einklemmung und ohne Gangrän	2 (0.7%)	0 (0%)	0.00	0.00, NA	0.999
K55.1 Ischämische Darmstriktur	1 (0.3%)	0 (0%)	0.00	0.00, NA	0.948
K56.0 Paralytischer Ileus	6 (2.0%)	5 (1.7%)	0.79	0.21, 3.0	0.726
N99 Nierenversagen nach med. Maßnahmen	7 (2.3%)	6 (2.1%)	0.84	0.23, 3.0	0.787
R57 Schock, anderenorts nicht klassifiziert	5 (1.6%)	0 (0%)	0.00	0.00, NA	0.941
R65 SIRS	11 (3.6%)	4 (1.4%)	0.38	0.12, 1.2	0.099
U69.00 Nosokomiale Pneumonie	4 (1.3%)	0 (0%)	0.00	0.00, NA	0.917
U69.01 Nosokomiale Pneumonie, >48h nach Aufnahme	3 (1.0%)	0 (0%)	0.00	0.00, NA	0.999
N30.0 Cystitis	14 (4.6%)	5 (1.7%)	0.30	0.10, 0.90	0.031
N32.0 Blasenhalsobstruktion	1 (0.3%)	0 (0%)			
N35 Harnröhrenstriktur	4 (1.3%)	1 (0.3%)	0.26	0.03, 2.5	0.246
N45.9 Epididymitis ohne Abszess	3 (1.0%)	0 (0%)	0.00	0.00, NA	0.962
N99.1 Harnröhrenstriktur nach med. Maßnahmen	0 (0%)	1 (0.3%)			
R33 Harnverhaltung	2 (0.7%)	2 (0.7%)	1.1	0.15, 7.6	0.951
R39.0 Urinextravasation	3 (1.0%)	3 (1.0%)	0.52	0.05, 5.0	0.571
T81.0 Hämatom als Komplikation nach OP	11 (3.6%)	3 (1.0%)	0.28	0.08, 1.0	0.054
T81.2 Versehentliche Stich- oder Rissverletzung während einer OP	26 (8.5%)	3 (1.0%)	0.27	0.07, 1.1	0.064
T81.3 Aufreißen einer OP-Wunde	5 (1.6%)	8 (2.8%)	1.7	0.49, 6.1	0.391
T81.4 Infektion nach einen Eingriff	7 (2.3%)	5 (1.7%)	0.75	0.24, 2.4	0.634
T81.5 Fremdkörper, versehentlich belassen	1 (0.3%)	0 (0%)	0.00	0.00, NA	0.998
D62 Blutungsanämie	81 (26%)	34 (12%)	0.35	0.21, 0.57	< 0.001
J91 Pleuraerguss bei anderenorts klassifizierten Krankheiten	3 (1.0%)	1 (0.3%)	0.35	0.04, 3.4	0.367
J90 Pleuraerguss, anderenorts nicht klassifiziert	7 (2.3%)	3 (1.0%)	0.43	0.10, 1.8	0.239

N17.91 Akutes Nierenversagen, nicht näher bezeichnet : Stadium 1	52 (17%)	46 (16%)	0.96	0.57, 1.6	0.868
N17.92 Akutes Nierenversagen, nicht näher bezeichnet : Stadium 2	7 (2.3%)	8 (2.8%)	1.2	0.44, 3.4	0.704
N17.93 Akutes Nierenversagen, nicht näher bezeichnet : Stadium 3	18 (5.9%)	5 (1.7%)	0.27	0.10, 0.78	0.016
N17.99 Akutes Nierenversagen, nicht näher bezeichnet : Stadium nicht näher bezeichnet	6 (2.0%)	5 (1.7%)	0.91	0.25, 3.3	0.882
K55.0 Akut: Darminfarkt, Dünndarmischämie, fulminante ischämische Kolitis	2 (0.7%)	0 (0%)	0.00	0.00, NA	0.958
A04.7 Enterokolitis durch Clostridium difficile	2 (0.7%)	0 (0%)	0.00	0.00, NA	0.999
I48 Vorhofflimmern und Vorhofflattern	37 (12%)	32 (11%)	0.93	0.55, 1.6	0.795
R19.80 Abdominales Kompartmentsyndrom	1 (0.3%)	0 (0%)	0.00	0.00, NA	0.946
T79.6 Kompartmentsyndrom	0 (0%)	1 (0.3%)	157,147,373	0.00, NA	0.987
U07.1 SARS-CoV-2-Infektion nachgewiesen	3 (1.0%)	0 (0%)			

Ergänzungstabelle 1b – Postoperative Behandlungen und Komplikationen (offen-chirurgisch vs. Roboter-assistiert)

**Behandlungen**	**Gesamter Zeitraum**				
	**Offen-chirurgisch**, n = 306 [1]	**RAL** [4], n = 78 [1]	**Differenz**	**95% CI** [2]	**p-Wert**
5-408.2 Drainage einer Lymphozele	1 (0.3%)	0 (0%)	0.00	0.00, NA	0.969
5-462 Anlegen eines Enterostomas (protektiv)	1 (0.3%)	0 (0%)	0.00	0.00, NA	0.999
5-536 Verschluss Narbenhernie	2 (0.7%)	0 (0%)	0.00	0.00, NA	0.981
5-541 Laparotomie / Eröffnung des Retroperitoneums	5 (1.6%)	3 (3.8%)	2.4	0.56, 10	0.236
5-896 Chirurgische Wundtoilette [Wunddebridement] mit Entfernung von erkranktem Gewebe an Haut und Unterhaut	3 (1.0%)	0 (0%)	0.00	0.00, NA	0.945
5-896.0 Chirurgische Wundtoilette [Wunddebridement] mit Entfernung von erkranktem Gewebe an Haut und Unterhaut: Kleinflächig	1 (0.3%)	0 (0%)	0.00	0.00, NA	1.000
5-896.1 Chirurgische Wundtoilette [Wunddebridement] mit Entfernung von erkranktem Gewebe an Haut und Unterhaut: Großflächig	2 (0.7%)	0 (0%)	0.00	0.00, NA	0.957
5-896.1 Chirurgische Wundtoilette [Wunddebridement] mit Entfernung von erkranktem Gewebe an Haut und Unterhaut: Großflächig, mit Einlegen eines Medikamententrägers	2 (0.7%)	0 (0%)	0.00	0.00, NA	0.957
5-916.a Anlage oder Wechsel eines Systems zur Vakuumtherapie	4 (1.3%)	0 (0%)	0.00	0.00, NA	0.943
5-916.a5 Anlage oder Wechsel eines Systems zur Vakuumtherapie: Tiefreichend subfaszial an der Bauchwand oder im Bereich von Nähten der Faszien bzw. des Peritoneums	2 (0.7%)	0 (0%)	0.00	0.00, NA	0.999
8-148 Therapeutische Drainage von anderen Organen und Geweben	3 (1.0%)	2 (2.6%)	2.7	0.44, 16	0.289
8-148.0 Therapeutische Drainage von anderen Organen und Geweben: Peritonealraum	1 (0.3%)	2 (2.6%)	9.2	0.68, 124	0.096
8-148.1 Therapeutische Drainage von anderen Organen und Geweben: Retroperitonealraum inkl. Psoas	2 (0.7%)	0 (0%)	0.00	0.00, NA	0.984
8-800.c Erythrozytenkonzentrat	57 (19%)	5 (6.4%)	0.30	0.12, 0.80	0.016
8-800.c0 Erythrozytenkonzentrat: 1 TE bis unter 6 TE	53 (17%)	5 (6.4%)	0.34	0.13, 0.89	0.028
8-800.c1 Erythrozytenkonzentrat: 6 TE bis unter 11 TE	2 (0.7%)	0 (0%)	0.00	0.00, NA	0.999
8-713 Maschinelle Beatmung und Atemunterstützung bei Erwachsenen	1 (0.3%)	0 (0%)	0.00	0.00, NA	0.965
8-771 Kardiale oder kardiopulmonale Reanimation	2 (0.7%)	0 (0%)	0.00	0.00, NA	0.999

Komplikationen

A40 Streptokokkensepsis	1 (0.3%)	0 (0%)	0.00	0.00, NA	0.985
A41 Sonstige Sepsis	10 (3.3%)	0 (0%)	0.00	0.00, NA	0.943
I21 Akuter Myokardinfarkt	4 (1.3%)	0 (0%)	0.00	0.00, NA	0.935
I26 Lungenembolie	2 (0.7%)	1 (1.3%)	2.0	0.14, 28	0.620
I63 Hirninfarkt	2 (0.7%)	0 (0%)	0.00	0.00, NA	0.992
I80 Thrombose, Phlebitis, Thrombophlebitis	5 (1.6%)	0 (0%)	0.00	0.00, NA	0.999
I82.2 Embolie und Thrombose der V. cava	1 (0.3%)	0 (0%)	0.00	0.00, NA	0.995
I82.9 Embolie und Thrombose nicht näher bezeichneter Venen	1 (0.3%)	0 (0%)	0.00	0.00, NA	0.994
J95.2 Akute pulmonale Insuffizienz	4 (1.3%)	0 (0%)	0.00	0.00, NA	1.000
J96.0 Akute respiratorische Insuffizienz	27 (8.8%)	2 (2.6%)	0.27	0.06, 1.2	0.080
K25.0 Ulcus ventriculi: Akut, mit Blutung	2 (0.7%)	0 (0%)	0.00	0.00, NA	1.000
K26.0 Ulcus duodeni: Akut, mit Blutung	2 (0.7%)	0 (0%)	0.00	0.00, NA	1.000
K65 Peritonitis	5 (1.6%)	0 (0%)	0.00	0.00, NA	0.999
K43.2 Narbenhernie ohne Einklemmung und ohne Gangrän	2 (0.7%)	0 (0%)	0.00	0.00, NA	0.981
K55.1 Ischämische Darmstriktur	1 (0.3%)	0 (0%)	0.00	0.00, NA	0.999
K56.0 Paralytischer Ileus	6 (2.0%)	0 (0%)	0.00	0.00, NA	0.942
N99 Nierenversagen nach med. Maßnahmen	7 (2.3%)	0 (0%)	0.00	0.00, NA	0.998
R57 Schock, anderenorts nicht klassifiziert	5 (1.6%)	0 (0%)	0.00	0.00, NA	0.970
R65 SIRS	11 (3.6%)	1 (1.3%)	0.35	0.04, 2.7	0.316
U69.00 Nosokomiale Pneumonie	4 (1.3%)	0 (0%)	0.00	0.00, NA	0.984
U69.01 Nosokomiale Pneumonie, >48h nach Aufnahme	3 (1.0%)	0 (0%)	0.00	0.00, NA	0.998
N30.0 Cystitis	14 (4.6%)	2 (2.6%)	0.35	0.07, 1.8	0.214
N32.0 Blasenhalsobstruktion	1 (0.3%)	0 (0%)	0.00	0.00, NA	0.987
N35 Harnröhrenstriktur	4 (1.3%)	1 (1.3%)	0.96	0.07, 12	0.973
R33 Harnverhaltung	2 (0.7%)	1 (1.3%)	2.0	0.18, 22	0.581
R39.0 Urinextravasation	3 (1.0%)	0 (0%)	0.00	0.00, NA	0.946
T81.0 Hämatom als Komplikation nach OP	11 (3.6%)	3 (3.8%)	1.1	0.29, 3.9	0.916
T81.2 Versehentliche Stich- oder Rissverletzung während einer OP	26 (8.5%)	0 (0%)	0.00	0.00, NA	0.994
T81.3 Aufreißen einer OP-Wunde	5 (1.6%)	1 (1.3%)	0.92	0.09, 9.1	0.944

T81.4 Infektion nach einen Eingriff	7 (2.3%)	0 (0%)	0.00	0.00, NA	0.925
T81.5 Fremdkörper, versehentlich belassen	1 (0.3%)	0 (0%)	0.00	0.00, NA	0.995
D62 Blutungsanämie	81 (26%)	8 (10%)	0.25	0.11, 0.59	0.002
J91 Pleuraerguss bei anderenorts klassifizierten Krankheiten	3 (1.0%)	0 (0%)	0.00	0.00, NA	0.999
J90 Pleuraerguss, anderenorts nicht klassifiziert	7 (2.3%)	2 (2.6%)	1.1	0.23, 5.5	0.885
N17.91 Akutes Nierenversagen, nicht näher bezeichnet : Stadium 1	52 (17%)	12 (15%)	1.1	0.51, 2.6	0.748
N17.92 Akutes Nierenversagen, nicht näher bezeichnet : Stadium 2	7 (2.3%)	1 (1.3%)	0.50	0.04, 5.6	0.571
N17.93 Akutes Nierenversagen, nicht näher bezeichnet : Stadium 3	18 (5.9%)	1 (1.3%)	0.21	0.03, 1.6	0.129
N17.99 Akutes Nierenversagen, nicht näher bezeichnet : Stadium nicht näher bezeichnet	6 (2.0%)	1 (1.3%)	0.98	0.08, 12	0.986
K55.0 Akut: Darminfarkt, Dünndarmischämie, fulminante ischämische Kolitis	2 (0.7%)	0 (0%)	0.00	0.00, NA	0.999
A04.7 Enterokolitis durch Clostridium difficile	2 (0.7%)	0 (0%)	0.00	0.00, NA	0.960
I48 Vorhofflimmern und Vorhofflattern	37 (12%)	7 (9.0%)	0.80	0.32, 2.0	0.625
R19.80 Abdominales Kompartmentsyndrom	1 (0.3%)	0 (0%)	0.00	0.00, NA	0.999
U07.1 SARS-CoV-2-Infektion nachgewiesen	3 (1.0%)	0 (0%)	0.00	0.00, NA	0.486

[1] n (%); Mittelwert (SD); Median [25%-75]
[2] CI = Konfidenzintervall
[3] Minimalinvasiv = konventionell-laparoskopisch und Roboter-assistiert
[4] RAL = Roboter-assistiert
